# Beyond the Bench: Of Mice and Women

**DOI:** 10.1289/ehp.115-a249

**Published:** 2007-05

**Authors:** Tanya Tillett

Scientists are making strides every day in breast cancer research through the use of mouse models in the lab. These investigations offer a better understanding of the cell processes that lead to development of the disease. Translating the results of this research into information that can be understood and used by a lay audience can be difficult, but the Community Outreach and Translation Core (COTC) of the NIEHS-funded Bay Area Breast Cancer and the Environment Research Center (BABCERC) has created a DVD for the public that offers simple, understandable descriptions and updates on various mouse models being used in breast cancer research.

Based on a presentation developed by Mary Helen Barcellos-Hoff, a senior scientist at the Lawrence Berkeley National Laboratory and a BABCERC co-investigator, and produced by Michael Hoff Productions, the DVD *Of Mice and Women: Modeling Breast Cancer and the Environment* is a 35-minute information session designed for a range of audience members from high school science students to breast cancer advocates. The DVD was originally created for distribution to the other three NIEHS-funded breast cancer centers to help teach communities about the research process. That way, when study results are published, the average community member can put them into an understandable context.

The video describes why mouse models are ideal for this particular field of research. For example, Barcellos-Hoff notes that mice and humans have similar mammary gland development and structure. This commonality allows investigators to manipulate rodent genes in search of answers to questions regarding the mechanisms behind breast cancer development, including analysis of the effects of environmental exposures. The video also presents information on models such as protein mapping. Study of specific functions at specific protein locations in mouse cells gives researchers insight into which genetic mutations lead to the development of breast cancer.

The COTC has also produced an accompanying scientific glossary that summarizes the information discussed in the DVD and defines terms used in the field of breast cancer research. The free DVD has become a popular educational tool used for a variety of audiences around the country, says Janice Barlow, executive director of Zero Breast Cancer, the nonprofit organization that leads the COTC. Its message may soon be delivered to more. According to Barlow, a California television station has expressed interest in broadcasting it, and there are plans to produce more copies. Above all, Barlow states that the ultimate goal in supporting research such as that featured in the DVD is to help ensure that desired health benefits and outcomes are achieved—namely, prevention of breast cancer, a decrease in breast cancer incidence and mortality, and a reduction of environmental exposures in our communities. For more information on the DVD, visit http://www.zerobreastcancer.org/ or e-mail janiceb@zerobreastcancer.org.

## Figures and Tables

**Figure:**
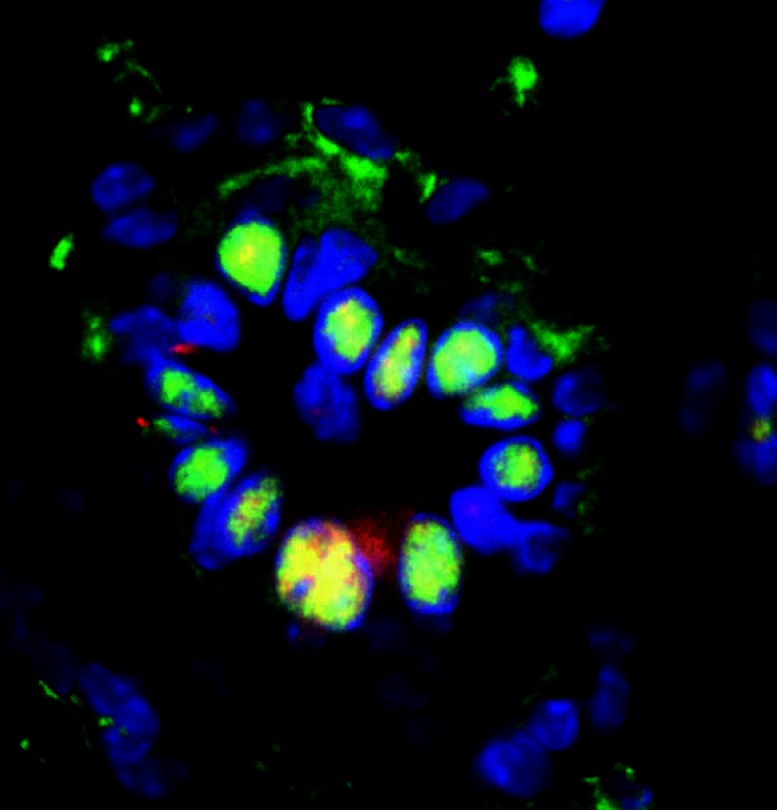
Normal mammary gland in cross-section

